# Saffron and crocin improved appetite, dietary intakes and body composition in patients with coronary artery disease

**DOI:** 10.15171/jcvtr.2017.35

**Published:** 2017-12-30

**Authors:** Nasim Abedimanesh, S. Zahra Bathaie, Saeed Abedimanesh, Behrooz Motlagh, Ahmad Separham, Alireza Ostadrahimi

**Affiliations:** ^1^Nutrition Research Center, Tabriz University of Medical Sciences, Tabriz, Iran; ^2^Department of Clinical Biochemistry, Tarbiat Modares University, Tehran, Iran; ^3^Faculty of Medicine, Department of Biochemistry & Nutrition, Zanjan University of Medical Sciences, Zanjan, Iran; ^4^Cardiovascular Research Center, Shahid Madani Heart Hospital, Tabriz University of Medical Sciences, Tabriz, Iran

**Keywords:** Appetite, Coronary Artery Disease, Crocin, Fat Mass, Saffron, Waist Circumference

## Abstract

***Introduction:*** Central obesity is an independent risk factor for coronary artery disease (CAD). It can increase cardio-metabolic risks through hypertension, hyperlipidemia and insulin resistance. Saffron and its bioactive compounds (crocin and crocetin) can modify some of metabolic disorders through multiple mechanisms. The aim of this study was to assess the efficacy of saffron and crocin on lipid profile, appetite, dietary intakes, anthropometric indices and body composition in patients with CAD.

***Methods:*** This 8 weeks randomized, double-blind, and placebo-controlled trial was conducted on 84 patients with CAD between the ages of 40 and 65 years old. Participants were randomly divided into groups to receive a daily supplement of 30 mg saffron aqueous extract (SAE) or 30 mg crocin or placebo. Appetite, dietary intake, anthropometry, body composition, biochemical analysis were assessed before and after the study.

***Results:*** In SAE and crocin group, anthropometric and some body composition variables revealed a pattern of improvement after intervention. Decrease in body mass index (BMI), waist circumference and fat mass values in SAE group was significantly more than crocin group (*P *< 0.001). There was no significant difference at the end of study in lipid profile parameters. Both SAE and crocin yielded significant decrease in energy and dietary intake mean values (*P *< 0.001 and *P *= 0.046), while it remained unchanged in the placebo group, also the appetite decreased significantly in SAE and crocin group (*P *< 0.001 and *P *= 0.029, respectively).

***Conclusion:*** The results of present study regarding anti-obesity feature of SAE and crocin in patients with CAD was promising. However the SAE was better in appetite suppressing, dietary intake and central obesity reduction.

## Introduction

Coronary artery disease (CAD) is one of the most important causes of death in the developed world.^1^ Also it is common health problem among adult Iranian.^2^ Recently the prevalence of risk factors associated with CAD such as adherence to poor nutritional behaviors, low physical activity and epidemic of obesity is high in developing countries and also in Iran.^2,3^ Although the obesity especially abdominal form can increase cardio-metabolic risks through hypertension, type 2 diabetes, hyperlipidemia, insulin resistance and left ventricular hypertrophy^4-6^ it is known as an independent risk factor for CAD according to some investigations. Adipose tissue and adipocytes are recognized as a source which secret several substances with biological activity, known as “adipokines”.^7,8^ They seem involved in the regulation of many physiological processes, such as appetite regulation, energy balance, lipid metabolism, blood pressure, insulin sensitivity and inflammation.^9^

Obesity is considered as an illness and overweight causes wide range of diseases.^10^ The global rate of overweight and obesity is increasing.^11^ In line with the global trend, this problem involved Asian countries, especially Iran.‏ The prevalence of overweight among Iranian by the World Health Organization (WHO)‏ reports was over 50% which expected to raise this prevalence during the period of 2005-2015.^12^

There are multiple approaches and strategies to deal with obesity including lifestyle modifications (adherence to healthy diet, weight loss and increasing physical activity), pharmacotherapy and surgery as a last resort.^13^ Almost all anti-obesity drugs adversely affected cardio-vascular function and also have other side effects, so the compliance of these drugs are low.^14^ The usage of plant based supplements for appetite control and increasing resting metabolism is common among obese subjects. They believe these supplements are foods, natural and safe, rather than medicines.^15^

Saffron (*Crocus sativus* Linn) has been used as an important dietary ingredient in different parts of the world since ancient times. It has also been applied in traditional medicine in the treatment of various kinds of illnesses including inflammatory and neurodegenerative disorders.^16^ The pharmacological activities of saffron are attributed to many of its active constituents such as volatile agents (e.g., safranal), bitter principles (e.g., picrocrocin), and dye materials (e.g., crocetin and crocins). The crocin is unique water-soluble carotenoid (cis and trans glucosyl ester of crocetin).^16^ Saffron and its main constituent; crocin have been shown to possess antidiabetic,^17^ antihyperlipidemic^18^ and hypotensive^19^ activities, on the other hand, some evidences indicated that saffron can enhance satiety and promote weight loss.^18,20,21^ So saffron and its bioactive compounds can modulate some metabolic disorders through multiple mechanisms.

The present randomized clinical trial was designed to assess the efficacy of saffron and its main carotenoid, crocin on lipid profile, appetite level, dietary intakes, anthropometric indices and body composition in patients with coronary artery disease.

## Methods and Materials

### Study population

Eighty-four outpatients with CAD recruited from the heart clinic of Shahid Madani Cardiovascular hospital in Tabriz, Iran. CAD (more than 50% stenosis) was documented by angiography. In designing of the study, we considered a confidence interval 95% and power of 90% with a two-sided test with α = 0.05 (type I error) and mean and standard deviation (SD) difference for body weight change in Gout et al.^20^ The number of subjects was 25 per group. Given an anticipated dropout rate of 10%, we set the enrollment target at 28 subjects. Included subjects were male and females aged 40–65 years. All patients took their usual medication related to their cardio-metabolic disorder (hypertension, hyperlipidemia etc) without any alteration until the end of the study. Hypertension was defined as systolic and/or diastolic blood pressure (DBP) ≥150/90 mm Hg or receiving anti-hypertensive medications. Inclusion criteria included the following: no history of any autoimmune disease, malignancy, insulin therapy, no pregnancy or nursing, no allergy to saffron products, no following diet therapy during last one year, no usage of agents which modulate appetite, weight loss or antidepressant pills in chemical or herbal form and no psychotherapy. Patients with heart attack or surgery were excluded from the study. All participants signed a written informed consent agreement.

### Treatment capsules

Saffron aqueous extract (SAE) was prepared by maceration method^22^ and crocin was extracted and purified by chromatography described in our previous report.^23^ Saffron stigmas were purchased from Ghaenat farmlands in Khorasan, Iran. In order to maintain the blind, identical capsules in same shape, weight, and color were filled with 30 mg of SAE/crocin plus vehicle (corn starch). Placebo capsules were also filled with the same vehicle.

### Study design

This randomized double-blind, placebo-controlled clinical trial conducted between January 2016 and November 2016. Capsules were administered by a blinded clinical researcher to blinded patients. Subjects were asked to take one capsule containing SAE, crocin or placebo after lunch with sufficient water for eight weeks. Two visits were conducted: one prior to the study to collect baseline data and then one at the end of week 8 to complete questionnaires for food records, appetite, anthropometry, body composition and lipid profile measurements. Participants were asked not to change their usual dietary intake and physical activity during the study. Compliance with treatment was assessed by capsules counts. For this, all participants returned their container at the end of the each 4-week interval.

### Dietary intake and appetite assessment

Dietary intakes were assessed by using food records completed for 3 days (2 week day and 1 weekend day) a week before intervention as well as at the end of study. Energy and macronutrient compositions were analyzed using the Nutritionist IV for Windows software program (The Hearst Corporation, San Bruno, CA).

Participant’s appetite level prior to lunch was assessed before and at the end of the intervention, by means of visual analog scales (VASs). Scoring was made on a 100-mm marked line between “no appetite” at one end and “uncontrollable appetite” at the other, with low, average, high, and very high points in between.

### Anthropometric measurements and body composition

Height was measured to the nearest 0.5 cm using a wall-mounted Stadiometer in standing position without shoes. Weight and body composition including body fat mass (FM), fat free mass (FFM), and the percentage of FM were measured using body composition analyzer (Tanita BC-418, Tanita Corporation of America, Inc.). Body mass index (BMI) was calculated as body weight (kg) divided by the square of height (m2). Waist and hip circumference were measured to the nearest 0.1 cm in duplicate to the nearest millimeter using a spring-loaded tape measure.

Dietary intake, appetite assessment, anthropometric and body composition measurements were obtained twice, before and after intervention.

### Biochemical analysis

After an overnight fasting period (10-12 hours), blood samples were obtained at baseline and at week 8 of the intervention. Plasma concentrations of glucose, triglyceride (TG), total cholesterol (TC), high-density lipoprotein cholesterol (HDL-C), low-density lipoprotein cholesterol (LDL-C) were measured by a colorimetric method using Pars Azmoon kits (Pars azmoon.co, Tehran, Iran) with an auto-analyzer (Hitachi, Japan).

### Statistical analysis

Statistical analysis was performed using SPSS software version 17.0 (SPSS Co., Chicago, IL, USA). The baseline characteristics between groups were compared using Fisher exact test. Analysis of variance (ANOVA) and Tukey test was used to compare baseline values, respectively for homogenous and heterogeneous groups according to their variances. A paired *t* test was used for comparison the mean differences (95% CI) within group. Analysis of covariance (ANCOVA) was used to test the difference between study groups after intervention, adjusting for baseline measurements, age and sex. In all analysis, *P* < 0.05 was considered as statistically significant.

## Results

### Participants

**Figure 1:**
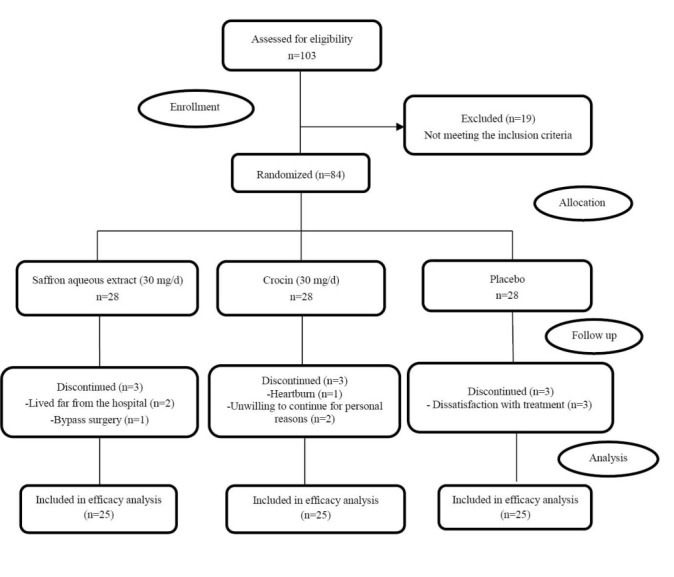
Flowchart of the trial.

Eligible subjects (n = 84) were randomly assigned by using a computer-generated random numbers method by the statistical analyst to three study groups. Of these, 75 (about 89%) completed the entire period of study (Figure 1). About 80% of participants had moderate physical activity. Demographic and baseline clinical characteristics of participants addressed in Table 1.

**Table 1:** Demographic and baseline clinical characteristics of participants (n = 75)

**Variables** ^a^	**SAE (n=25)**	**Crocin (n=25)**	**Placebo (n=25)**	***P*** ** value** ^#^
Age, y	56.04±7.55	53.36±5.94	56.32±5.91	0.214
Sex, male	14 (56%)	13 (52%)	12 (48%)	0.957
CAD family history	13 (52%)	11 (44%)	9 (36%)	0.569
Smoking	1 (4%)	5 (20%)	5 (20%)	0.059
Hypertension	14 (56%)	18 (72%)	14 (56%)	0.441
Diabetes mellitus	3 (12%)	5 (20%)	5 (20%)	0.799
Hypoglycemic drugs	3 (12%)	5 (20%)	5 (20%)	0.799
Antihypertensive drugs	18 (72%)	18 (72%)	14 (56%)	0.420
Hypolipidemic drugs	16 (64%)	14 (56%)	16 (64%)	0.872

SAE: Saffron Aqueous Extract, CAD: Coronary Artery Disease ^#^ *P* for between-group comparison with ANOVA and Fisher exact test where appropriate ^a^Numeric variables expressed as mean ± SD and Categorical variables expressed as n (%).

Table 2 summarizes pre and post-intervention changes in the anthropometric and body composition variables. In SAE and crocin group all variables revealed a pattern of improvement after intervention. However FFM remained unchanged in crocin group (*P =* 0.582). Between-group comparison showed the baseline mean values were same in all variables of Table 2, but after intervention, there was significant difference between study groups (P<0.001 or *P =* 0.001). After adjustment for age and sex the same results were obtained. Weight loss was significantly higher in SAE group in comparison to crocin group (MD= -1.37, CI: -2.173 to -0.571, *P* < 0.001). Also, decrease in BMI, waist circumference, fat mass and fat free mass mean values in SAE group was significantly more than crocin group (*P* < 0.001, 0.001, 0.016 and *P* < 0.001, respectively).

**Table 2:** Anthropometric and body composition measurements in study groups

**Variables**	**SAE (n=25)**	**Crocin (n=25)**	**Placebo (n=25)**	***P***	***P*** ^#^
Body weight, kg					
Baseline	83.60±12.66	79.68±10.30	78.20±10.78	0.224 ^a^	
Week 8	81.29±12.66	78.79±10.30	78.24±10.49	<0.001^b^	<0.001
Within group MD (95% CI) *P* value ^c^	-2.31(-2.88 to -1.74) <0.001	-0.89(-1.33 to -0.45) <0.001	0.04(-0.34 to 0.43) 0.818		
BMI, kg/m^2^					
Baseline	28.64±2.23	27.92±2.57	28.05±2.89	0.576 ^a^	
Week 8	27.84±2.32	27.62±2.53	28.08±2.88	<0.001^b^	<0.001
Within group MD (95% CI) *P* value ^c^	-0.79(-0.98 to -0.61) <0.001	-0.29(-0.45 to -0.14) 0.001	0.03(-0.11 to 0.16) 0.674		
Waist, cm					
Baseline	95.00±12.85	92.84±9.13	91.84±10.73	0.642 ^a^	
Week 8	92.68±13.03	91.42±8.94	92.13±10.88	<0.001^b^	<0.001
Within group MD (95% CI) *P* value ^c^	-2.32( -2.76 to -1.88) <0.001	-1.42(-1.83 to -1.01) <0.001	0.29(-1.18 to 1.76) 0.686		
WHR					
Baseline	0.92±0.07	0.90±0.06	0.92±0.05	0.501^a^	
Week 8	0.90±0.07	0.89±0.06	0.91±0.04	0.001^b^	0.002
Within group MD (95% CI) *P* value ^c^	-0.01(-0.02 to -0.01) <0.001	-0.01(-0.02 to -0.01) <0.001	-0.003(-0.007 to 0.001) 0.175		
FM, kg					
Baseline	26.61±4.23	25.78±7.39	25.26±5.52	0.715 ^a^	
Week 8	25.06±4.42	24.96±7.40	25.23±5.63	<0.001^b^	<0.001
Within group MD (95% CI) *P* value ^c^	-1.55(-1.99 to -1.11) <0.001	-0.82(-1.22 to -0.43) <0.001	-0.03(-0.27 to 0.20) 0.768		
Fat %					
Baseline	32.16±4.83	32.29±7.60	32.38±6.14	0.992^a^	
Week 8	31.14±5.05	31.58±7.66	32.31±6.14	<0.001^b^	<0.001
Within group MD (95% CI) *P* value ^c^	-1.02(-1.38 to -0.65) <0.001	-0.71(-1.06 to -0.36) <0.001	-0.07(-0.23 to 0.08) 0.341		
FFM, kg					
Baseline	56.99±10.98	53.89±8.99	52.94±9.25	0.316^a^	
Week 8	56.23±10.97	53.83±8.87	53.01±9.07	<0.001^b^	<0.001
Within group MD (95% CI) *P* value ^c^	-0.76 (-1.03 to -0.49) <0.001	-0.07(-0.31 to 0.18) 0.582	0.08(-0.10 to 0.25) 0.373		

SAE: Saffron Aqueous Extract, BMI: Body Mass Index, WHR: Waist to Hip Ratio, FM: Fat Mass, FFM: Fat Free Mass, MD: Mean Difference, CI: confidence interval ^a^Data are presented as means±SD. Statistical analyses are performed by means of one way ANOVA. ^b^Data are presented as means±SD. Statistical analyses are performed by means of ANCOVA adjusted for baseline measurements. ^c^Within group mean difference (95% confidence interval) based on paired t-test. ^#^ *P* value based on ANCOVA adjusted for baseline measurements, age and sex.

According to Table 3, the biochemical variables were similar among the study groups at baseline. Intervention did not affect these variables significantly. Between groups comparison revealed that there was no significant difference at the end of study. But after adjustment for age and sex, LDL to HDL ratio was significantly different among study groups (*P* = 0.040). Decrease in mentioned ratio between SAE and placebo group was significant according to Sidak test (MD= -0.391, CI: -0.691 to -0.091, *P* = 0.012).

**Table 3:** Fasting blood glucose and lipid profiles in study groups

**Variable**	**SAE (n=25)**	**Crocin (n=25)**	**Placebo (n=25)**	***P*** ** value**	***P*** ** value** ^#^
FBS (mg/dL)					
Baseline	107.25±44.14	123.11±59.31	100.05±25.74	0.189 ^a^	
Week 8	100.85±26.49	117.63±56.48	98.26±23.72	0.726^b^	0.614
Within group MD (95% CI) *P* value ^c^	-6.40(-17.35 to 4.55) 0.236	-5.47(-23.81 to 12.86) 0.538	-1.79(-0.54 to 0.00) 0.052		
Triglycerides (mg/dL)					
Baseline	200.05±74.08	182.37±87.27	171.26±48.78	0.365^a^	
Week 8	193.05±60.44	192.32±101.00	179.11±38.36	0.732^b^	0.805
Within group MD (95% CI) *P* value ^c^	-7.00(-37.75 to 23.75) 0.639	9.95(-18.29 to 38.18) 0.469	7.85(0.36 to 21.32) 0.053		
Total Cholesterol (mg/dL)					
Baseline	176.50±38.24	166.26±32.66	172.78±39.19	0.611^a^	
Week 8	171.90±36.38	172.11±33.87	181.05±42.33	0.233^b^	0.270
Within group MD (95% CI) *P* value ^c^	-4.60(-16.11 to 6.91) 0.413	5.84(-6.10 to 17.78) 0.318	8.26(-1.58 to 18.10) 0.095		
HDL-C (mg/dL)					
Baseline	42.35±6.74	45.84±6.52	45.89±7.58	0.127 ^a^	
Week 8	45.70±9.06	47.84±8.33	45.53±7.86	0.330^b^	0.157
Within group MD (95% CI) *P* value ^c^	3.35(-0.42 to 7.12) 0.079	2.00(-0.99 to 4.99) 0.177	-0.37 (-2.68 to 1.94) 0.742		
LDL-C (mg/dL)					
Baseline	94.10±35.40	81.31±28.47	93.10±32.09	0.299 ^a^	
Week 8	89.20±32.34	83.21±26.23	98.42±36.21	0.300^b^	0.278
Within group MD (95% CI) *P* value ^c^	-4.90(-14.20 to 4.40) 0.284	1.89(-9.23 to 13.02) 0.725	5.31(-4.44 to 15.07) 0.267		
LDL/HDL					
Baseline	2.23±0.78	1.80±0.63	2.08±0.76	0.112 ^a^	
Week 8	1.96±0.72	1.80±0.67	2.18±0.76	0.091^b^	0.040
Within group MD (95% CI) *P* value ^c^	-0.27(-0.54 to 0.00) 0.052	0.00(-0.19 to 0.19) 1.000	0.09(-0.11 to 0.30) 0.354		
VLDL, (mg/dL)					
Baseline	45.10±14.92	40.47±18.88	41.74±14.85	0.587 ^a^	
Week 8	43.65±12.50	42.21±20.89	43.16±13.33	0.750^b^	0.814
Within group MD (95% CI) *P* value ^c^	-1.45(-7.69 to 4.79) 0.632	1.74(-3.93 to 7.40) 0.528	1.42(-0.45 to 3.30) 0.129		

SAE: Saffron Aqueous Extract, FBS: fasting blood sugar, MD: Mean Difference, CI: confidence interval ^a^Data are presented as means±SD. Statistical analyses are performed by means of one way ANOVA. ^b^Data are presented as means±SD. Statistical analyses are performed by means of ANCOVA adjusted for baseline measurements. ^c^Within group mean difference (95% confidence interval) based on paired t-test. ^#^P-value based on ANCOVA adjusted for baseline measurements, age and sex.

Table 4 demonstrated the results of between group and within group comparison analysis on energy, macronutrients intake and appetite level variables. At baseline, all variables were similar except in protein intake and appetite variables (*P* < 0.001 or *P* = 0.008), but mentioned parameters were not significantly different between SAE and crocin group (*P* > 0.05). Paired *t* test comparisons revealed that when compared to baseline mean values, both SAE and crocin interventions yielded significant decrease in energy and dietary intake mean values (*P* < 0.001 and *P* = 0.046), while it remained unchanged in the placebo group, also the feeling of hunger decreased significantly in SAE and crocin groups (*P* < 0.001 and 0.029, respectively). There was significant difference between study groups in the mean value of energy, carbohydrate and protein intake at the end of study, before and after adjustment for age and sex (*P* < 0.001). According to Sidak test, the SAE group revealed significant difference in comparison to crocin and placebo group (*P* < 0.05) in these variables. Comparison between SAE and crocin group showed that, mean difference of energy, carbohydrate and protein intake was 85.18 kcal (*P* = 0.012), 13.78 (*P* = 0.004) and 4.71 g (*P* < 0.001) respectively. Feeling of hunger in SAE group decreased significantly compared to crocin group (MD= -2.384, CI: -4.229 to -0.539, *P* = 0.007). However, feeling of fullness and satiety in SAE group increased dramatically compared to crocin (MD= 1.741, CI: 0.628 to 2.855, *P* = 0.001 and MD = 1.526, CI: 0.126 to 2.926, *P* = 0.028, respectively).

**Table 4:** Energy, dietary intake and appetite parameters in study groups

**Variable**	**SAE (n=25)**	**Crocin (n=25)**	**Placebo (n=25)**	***P value***	***P value*** ^#^
Energy (kcal/d)					
Baseline	2527.35±234.12	2446.26±251.61	2385.97±180.68	0.089 ^a^	
Week 8	2356.91±280.31	2363.75±263.88	2372.06±149.05	<0.001^b^	<0.001
Within group MD (95% CI) *P* -value^c^	-170.44(-227.31 to -113.57) <0.001	-82.51(-113.21 to -51.81) <0.001	-13.92(-44.16 to 16.33) 0.352		
Carbohydrate (g/d)					
Baseline	373.86±44.00	364.15±48.67	360.26±28.82	0.492 ^a^	
Week 8	346.18±51.00	350.40±47.69	358.36±27.07	<0.001^b^	<0.001
Within group MD (95% CI) *P* -value ^c^	-27.69(-35.61 to -19.76) <0.001	-13.75(-18.65 to -8.85) <0.001	-1.90(-6.46 to 2.66) 0.397		
Protein (g/d)					
Baseline	70.76±8.99	67.61±5.61	64.19±6.74	0.008 ^a^	
Week 8	62.96±7.74	65.29±5.59	63.82±6.18	<0.001^b^	<0.001
Within group MD (95% CI) *P* -value ^c^	-7.79(-10.43 to -5.16) <0.001	-2.32(-3.19 to -1.44) <0.001	-0.37 (-1.17 to 0.43) 0.347		
Total fat (g/d)					
Baseline	83.21±10.52	79.91±11.32	76.46±9.62	0.083 ^a^	
Week 8	80.04±11.12	77.22±11.60	75.22±8.14	0.802^b^	0.836
Within group MD (95% CI) *P* -value ^c^	-3.17(-6.28 to -0.05) 0.046	-2.69(-3.71 to -1.66) <0.001	-1.24(-3.57 to 1.10) 0.285		
Feelings of fullness (mm)^§^					
Baseline	41.12±4.11	42.84±2.03	48.00±7.91	<0.001^a^	
Week 8	44.40±2.86	44.20±2.14	48.68±7.87	<0.001^b^	<0.001
Within group MD (95% CI) *P* -value ^c^	3.28(2.45 to 4.11) <0.001	1.36(0.72 to 2.00) <0.001	0.68(0.10 to 1.26) 0.024		
Feelings of satiety (mm)^§^					
Baseline	41.64±4.84	42.44±2.63	49.68±7.07	<0.001^a^	
Week 8	45.12±4.75	44.36±3.21	49.64±7.27	<0.001^b^	<0.001
Within group MD (95% CI) *P* -value ^c^	3.48(2.65 to 4.31) <0.001	1.92(0.95 to 2.88) <0.001	-0.04(-0.72 to 0.64) 0.904		
Feelings of hunger (mm)^§^					
Baseline	46.20±5.48	49.28±3.55	42.36±5.65	<0.001^a^	
Week 8	42.88±4.62	47.80±4.22	42.28±5.58	0.001^b^	0.007
Within group MD (95% CI) *P* -value ^c^	-3.32(-4.54 to -2.09) <0.001	-1.48(-2.79 to -0.17) 0.029	-0.08(-0.81 to 0.65) 0.824		

SAE: Saffron Aqueous Extract, MD: Mean Difference, CI: confidence interval ^a^Data are presented as means±SD. Statistical analyses are performed by means of one-way ANOVA. ^b^Data are presented as means±SD. Statistical analyses are performed by means of ANCOVA adjusted for baseline measurements. ^c^Within group mean difference (95% confidence interval) based on paired *t* test. ^#^P-value based on ANCOVA adjusted for baseline measurements, age and sex, but for appetite parameters adjusted for baseline measurements, baseline energy intake and sex. ^§^Millimeters of VAS.

## Discussion

The results of the present study confirmed that both the SAE and crocin were effective in improving anthropometric indices, some parameters of body composition, energy, carbohydrate and protein intake in patients with CAD in comparison to placebo, although SAE had stronger effects than crocin. Both interventions could favorably alter the parameters of appetite, but the effect of SAE was significant. Lipid profile variables did not change significantly after 8 weeks.

Previously, in a clinical trial the effects of alcoholic extract of saffron on appetite, anthropometric parameters and body composition were investigated in overweight women.^20^ According to this study, saffron consumption induced satiating effect, reduced snacking and leading to weight loss. Unlike our study, in this trial energy, dietary intake measurements and lipid profile have not been addressed. According to our knowledge, this study is the first report to compare the effect of SAE and crocin on mentioned parameters in patients with coronary artery disease in a randomized, double blind, placebo-controlled design. In the present study, both SAE and crocin were well tolerated by the patients and did not interfere with clinical improvement; besides, no serious side effects were observed. This was consistent with the previous reports on the safety of saffron and crocin in humans.^24,25^ Participants in present study were overweight and mildly obese. Fullness and satiety feeling were significantly low compared to crocin and placebo group. Daily consumption of SAE created satiety and fullness feeling in consistent with Gout et al.^20^ The effect of crocin on appetite parameters was in a same trend with SAE and it was significant. Crocin modestly suppressed the appetite, energy and macronutrients intake after 8 weeks of intervention. However it should be noted the effects of SAE was stronger than crocin. Some other active ingredients (safranal) in addition to crocin may be involved in the effects of saffron on these properties. In fact, there are some known synergistic interactions between bioactive molecules within the saffron composition.^26^ Probably these are possible mechanisms for saffron stronger effects.

The effects of saffron and its active constituents on dietary intakes was not assessed before in human studies. So there is no information regarding their effects on macronutrients intake. In present investigation, dietary intake of carbohydrate and protein were comparable between study groups, while it was almost similar for fat intake between groups at the end of study. By conducting more studies in this regard we could be able to assess the exact effect of saffron and crocin on macronutrients intake.

Limited studies investigated the anti-obesity potentials of saffron. Mashmoul et al^18^ evaluated the effect of saffron’s ethanolic extract and its main carotenoid crocin in a rat model of high fat diet–induced obesity. Results showed that saffron extract could restrict appetite and food consumption. The anorexigenic effect of saffron was confirmed by Kianbakht et al.^21^ In this study the effects of saffron and crocin compared to sibutramine were measured in adult male Wistar rats and the results suggested that both saffron and crocin could reduce the body weight, food intake and blood leptin levels significantly compared to the control group and baseline and also the effects were comparable to sibutramine. An active component in saffron, solely or synergic with other ingredients can modulate neurotransmitter pathways especially serotonin reuptake^27,28^ and probably it can target appetite control centers and limit food intake. Previous human controlled trials have pointed to this property of saffron and decreased feeling of hunger was indicated as side effect of saffron treatment.^29,30^ In present study, waist circumference, waist to hip ratio, fat mass and fat percent decreased in both interventions in comparison to placebo, with a higher magnitude in the SAE group. Similarly, Mashmoul et al^18^ reported the significant reduction in total fat pad and its subscales in crocin group. In a rat model, six weeks treatment with crocetin (a natural carotenoid compound found in the stigmas of saffron) decreased visceral fat accumulation significantly. Saffron components could inhibit the activity of pancreatic lipase and reduce fat absorption.^31^ They also could increase fat metabolism, lipolysis and energy expenditure.^32^ Probably, through these mechanisms saffron and its components could affect fat mass and consequently abdominal obesity. Although according to the literature, a limited number of studies have addressed the anti-obesity effects of saffron. So further research into the underlying mechanism of saffron extract and crocin on anthropometric and body composition modification is needed.

Central fat distribution is more relevant in CAD risk compared to total fat mass.^33^ Waist circumference and waist to hip ratio (WHR) are determinants of central obesity.^34^ Thus any modification of these indices could prevent undesirable outcomes of CAD. Central obesity, is associated with dyslipidemia characterized by elevated TG, LDL-C and reduced HDL-C concentrations. Dyslipidemia is a main cardiovascular risk factor for CAD. Several investigations revealed that saffron extract and its components could improve lipid profile and prevent obesity complications. Lee et al^35^ confirmed the hypolipidemic effects of crocin and crocetin in hyperlipidemic mice, also in two other studies on quails the similar results were obtained.^36,37^ Lipid lowering property of both crocin and saffron on diet-induced hyperlipidemic rats was shown previously.^31,38^ According to Asdaq et al^38^ the high dose of saffron (100 mg/kg) was found to be significantly better than high dose of crocin (19.38 mg/kg) in falling TC, TG and LDL-C. Samarghandian et al^22^ study evaluated the effect of SAE on STZ-induced diabetic rats and its effects on serum lipid profiles and some other metabolic parameters. Against the findings of our study, the results indicated that saffron could increase the body weight in diabetic rats and also ameliorate lipid profile levels. Parallel with Samarghandian et al, the findings of Shirali et al,^17,39^ confirmed the hypolipidemic effects of SAE and also crocin on a model of diabetes mellitus. The mechanism proposed regarding hypolipidemic effects of saffron is that, crocin selectively could inhibits the activity of pancreatic lipase and result in malabsorption of fat and cholesterol.^31^ However the results of experimental studies in this regard were not unanimous. An investigation on high lipid feeding rabbits revealed that crocetin had no effect on plasma lipid pattern.^40^ A 4-week randomized clinical trial conducted on patients suffering major depression and evaluated the effects of saffron on depression and lipid profile.^41^ Consistent with our study they did not observe any lipid lowering property. Probably, use of different doses of saffron and crocin and non-identical duration of study in animal investigations, indicated the different results. In several clinical trials mentioned in this study, the participants were not homogenous. Study populations were overweight women, patients suffering from depression or CAD. So it may be responsible for conflicting results.

The limitations of our study were its little sample size, short duration and fix dose of saffron and crocin. Because several studies indicated that the effects of saffron and crocin were dose dependent.

## Conclusion

The results of present study regarding anti-obesity feature of SAE and crocin in patients with CAD was promising. However the SAE was better in appetite suppressing, dietary intake and central obesity reduction. We did not observe significant lipid lowering effect in interventional groups compared to placebo group. There were limited number of human studies addressing the effects of saffron and crocin on anti-obesity, appetite reducing and modification of lipid profile. In the other hand the results of animal studies were inconsistent in this regards. Therefore it is highly recommended that, these findings are confirmed in a larger sample size with longer duration and various doses.

## Ethical approval

The present study is registered in the Iranian Registry Clinical Trials (IRCT201512102017N26). The Ethics Committee of Tabriz University of Medical Sciences approved this study (TBZMED.REC.1394.739).

## Competing interests

All authors declare no competing financial interests exist.

## Acknowledgements

This trial was financially supported by a grant from Vice-chancellor for Research, Nutrition Research Center (grant No. 5.71.1220), Tabriz University of Medical Sciences. We would like to thank the Tarbiat Modares University that provided saffron and extraction supplies.
